# *Mycoplasma bovis* 5′-nucleotidase is a virulence factor conferring mammary fitness in bovine mastitis

**DOI:** 10.1371/journal.ppat.1012628

**Published:** 2024-11-12

**Authors:** Aga E. Gelgie, Peleg Schneider, Christine Citti, Emilie Dordet-Frisoni, Barbara E. Gillespie, Raúl A. Almeida, Getahun E. Agga, Yaa Serwaah Amoah, Nahum Y. Shpigel, Oudessa Kerro Dego, Inna Lysnyansky

**Affiliations:** 1 Department of Animal Science, The University of Tennessee, Knoxville, Tennessee, United States of America; 2 The Koret School of Veterinary Medicine, The Robert H. Smith Faculty of Agriculture, Food and Environment, The Hebrew University of Jerusalem, Rehovot, Israel; 3 Interactions Hôtes-Agents Pathogènes (IHAP), Université de Toulouse, INRAE, ENVT, Toulouse, France; 4 Food Animal Environmental Systems Research Unit, Agricultural Research Service, United States Department of Agriculture, Bowling Green, Kentucky, United States of America; 5 Mycoplasma Unit, Department of Avian Diseases, Kimron Veterinary Institute, Beit Dagan, Israel; Miami University, UNITED STATES OF AMERICA

## Abstract

Nucleases and 5′ nucleotidase (5′-NT) play essential roles in cell biology and are often associated with bacterial virulence. In *Mycoplasma* spp., which have limited metabolic capacities and rely on nutrient availability, these enzymes are of significant importance for nucleotide salvage. This study explores the potential role of 2 membrane-associated lipoproteins, the major nuclease MnuA and 5′-NT, in *Mycoplasma bovis* mastitis. Mutants deficient in MnuA (mnuA::Tn) and in 5’-NT (0690::Tn) were identified through genome-wide transposon mutagenesis of *M*. *bovis* PG45 type strain and their fitness and virulence were assessed both *in vitro*, in axenic medium, and *in vivo*, using murine and cow mastitis models. The mnuA::Tn mutant demonstrated reduced nuclease activity, while 0690::Tn exhibited slow log-phase growth and impaired hydrolase activity towards nucleotides as well as deoxynucleotides (dAMP and dGMP). In comparison to the parent strain, the 0690::Tn mutant displayed markedly reduced fitness, as evidenced by a significant decrease or even absence in post-challenge mycoplasma counts in murine and cow mammary tissues, respectively. Moreover, the 0690::Tn mutant failed to induce mastitis in both experimental models. Conversely, the mnuA::Tn mutant induced inflammation in murine mammary glands, characterized by neutrophil infiltration and increased expression of major inflammatory genes. In cows, the mnuA::Tn was able to cause an increase in somatic cell counts in a manner comparable to the wild type, recruit neutrophils, and induce mastitis. Collectively, these findings provide complementary insights, revealing that disruption of 5′-NT significantly attenuated *M*. *bovis* pathogenicity, whereas a MnuA-deficient mutant retained the ability to cause mastitis.

## Author summary

There is an increasing recognition of the crucial role that genes involved in the metabolic pathways play in bacterial pathogenicity. Although these genes are not traditionally classified as virulence factors, they may confer survival advantages and contribute to bacterial fitness and virulence. This is particularly relevant for mycoplasmas, which lack many essential biosynthesis pathways and for which nutrient availability and acquisition are critical for successful host colonization, survival, and infection. In this study, we generated *Mycoplasma bovis* mutants deficient in the major surface nucleases MnuA and 5′-nucleotidase (5′-NT) using random transposon mutagenesis. We evaluated these mutants in both *in vitro* and *in vivo* murine and cow models. Our findings unveiled that *M*. *bovis* 5′-NT, rather than MnuA, plays a pivotal role in pathogen survival, fitness, and virulence in bovine mastitis. The structural resemblance between *M*. *bovis* 5′-NT and other bacterial 5′-NT, which are known for their significance as virulence factors and their impact on host immune responses, underscores its potential importance. Targeting this gene could offer a promising strategy for therapeutic interventions.

## Introduction

*Mycoplasma bovis* belongs to the class *Mollicutes*, a fastidious group of bacteria that is characterized by the lack of a cell wall, a reduced genome, limited metabolic capabilities and numerous nutritional requirements [1]. Despite these limitations, *M*. *bovis* is a successful pathogen causing a variety of diseases in bovines resulting in significant economic losses [2]. For successful survival and continuous multiplication, mycoplasma must compete with its host for nutrition; hence nutritional resources become a focal point.

Nucleotides, encompassing purines and pyrimidines along with their associated metabolic products, hold indispensable importance for all living organisms. They play crucial roles in vital cellular functions, including nucleic acid synthesis, DNA replication, RNA transcription, DNA repair, energy storage and metabolism, and cell signaling [3–5]. Additionally, nucleotides serve as constituents of essential coenzymes and assume key roles as activated intermediates in lipid and carbohydrate synthesis [6]. Due to their critical functions, nucleotides and their metabolites affect bacterial pathogenesis. Indeed, various genes involved in nucleotide biosynthesis are essential for bacterial growth, survival within the host, especially during intracellular replication, and for virulence (reviewed in [7]). Furthermore, disruptions in nucleotide biosynthesis may induce antibiotic persistence, thereby influencing antibiotic efficacy and contributing to antibiotic treatment failure [8,9].

In most bacteria, nucleotides are synthesized through both *de novo* and salvage pathways. However, the majority of mycoplasmas lack the biosynthetic machinery for *de novo* synthesis [10,11] and instead rely on pre-formed nucleobases or nucleosides, sourced either within the cell or transported from the external environment, to construct nucleotides. Nucleic acids serve as the primary source for the uptake of nucleotide precursors [12], and extracellular DNA (e-DNA) has been identified as a limiting nutrient for the proliferation of *M*. *bovis* in eukaryotic cells. Supplementation with e-DNA enhances bacterial growth, leading to a cytopathic effect due to H_2_O_2_ production [13]. The ability to non-specifically degrade and process nucleic acids is evidently advantageous for bacteria, and many *Mycoplasma* species express external membrane-associated nucleases [14]. A recent study demonstrated that the inactivation of *M*. *bovis* major membrane-associated nuclease MnuA (encoding by coding sequence (CDS) MBOVPG45_0215) abolished most of the nuclease activity in this pathogen [15]. Moreover, it was suggested that MnuA degrades the DNA component of neutrophil extracellular traps (NETs); NETs were observed *in vitro* in bovine neutrophils exposed to the *mnu*A mutant but not in neutrophils exposed to *M*. *bovis* wild type (WT) or the *mnuA*-complemented strain [16]. It is likely that the breakdown of structural DNA in NETs allows *M*. *bovis* to evade the host immune response and provides nutrients crucial for bacterial survival and pathogenesis. The degradation of NETs, as well as biofilm DNA backbone, by extracellular nucleases is a known bacterial strategy for survival fitness and virulence, documented in *Streptococcus pyogenes* [17], *Streptococcus pneumoniae* [18], *Vibrio cholerae* [19], *Staphylococcus aureus* [20,21], *Serratia marcescens* [22] and several mycoplasmas [23–26].

Another enzyme involved in nucleotide salvage is the 5′-nucleotidase (5′-NT), which catalyzes the hydrolytic dephosphorylation of 5′-ribonucleotides and 5′-deoxyribonucleotides into nucleosides/deoxynucleosides and orthophosphate. 5′-NTs are diverse and multifunctional enzymes widely distributed among mammals, plants, fungi, and bacteria [27]. 5′-NTs are classified based on cellular localization, hydrolysis mechanism, and nucleobase specificity [28]. Surface-located 5′-NTs, also known as ecto-5′-nucleotidases, have emerged as central regulators of extracellular balance between proinflammatory e-ATP and anti-inflammatory adenosine (Ado) molecules, both are known as important controllers of the immune cell functions acting via purinergic receptors present on various host immune cells [29,30]. Several extracellular nucleotidases have been identified in Gram-positive and Gram-negative bacteria, serving as important virulence factors that facilitate the pathogen’s evasion of the host immune defense [28,31]. In some pathogens, a synergy between 5′-NTs and secreted nucleases has been reported [20,32,33]. Recently, through a combination of genomic and metabolomic analyses, Masukagami et al. identified and annotated a putative 5′-NT (MBOVPG45_0690) in the genome of *M*. *bovis* [34].

*M*. *bovis* is a significant pathogen causing pneumonia, mastitis, arthritis, otitis media and other diseases in bovines. Infections associated with *M*. *bovis* are often chronic, resistant to antimicrobial therapy, and currently, there are no effective vaccines available [2]. Intramammary infection (IMI) by *M*. *bovis* can result in a wide spectrum of responses, ranging from minimal inflammation and negligible effects on milk production to severe clinical mastitis across all glands, potentially leading to permanent loss of production [35,36]. Even after the resolution of clinical mastitis, chronic subclinical mastitis or latent infections with intermittent shedding of the organism in milk can persist for months [37]. The persistence of the pathogen may be partially due to the ability of *M*. *bovis* to reside intracellularly. The successful establishment and persistence of *M*. *bovis* infections are driven by its virulence factors and the nature of the immune response elicited by the pathogen; however, both aspects remain poorly understood. Considering the important roles of exonucleases and 5′-NT play in fundamental cellular processes and their association with bacterial virulence, our study aimed to investigate the potential roles of MnuA and 5′-NT in the pathogenesis of *M*. *bovis* mastitis. Using genome-wide transposon (Tn) mutagenesis, we identified and characterized mutants in *mnuA* and MBOVPG45_0690. These mutant clones were further validated under *in vitro* condition, in axenic medium, and in *in vivo*, using mice and dairy cows *M*. *bovis* mastitis models.

## Results

### Identification of *M*. *bovis* PG45 mutants deficient in the major membrane nuclease MnuA and the 5′ nucleotidase

A library of 1700 individual mutants of the *M*. *bovis* PG45 was screened using MBOVPG45_0215 (*mnuA*) or MBOVPG45_0690 (5′-NT) gene-specific primers, coupled with mTn-specific primers, as detailed in the Materials and Methods section and S1 Table. The mnuA::Tn mutant, upon identification, exhibited a single mTn insertion in the *mnuA* gene, precisely 295 bp from the predicted start codon and the 0690::Tn mutant displayed an insertion located 1313 bp into the 5′-NT gene (S2 Table). The phenotypic features of these mutants along with their complemented strains are described below, both *in vitro* and *in vivo*.

### Domain structure and conserved amino acid residues in 5′ nucleotidase of *M*. *bovis*

**Fig 1:**
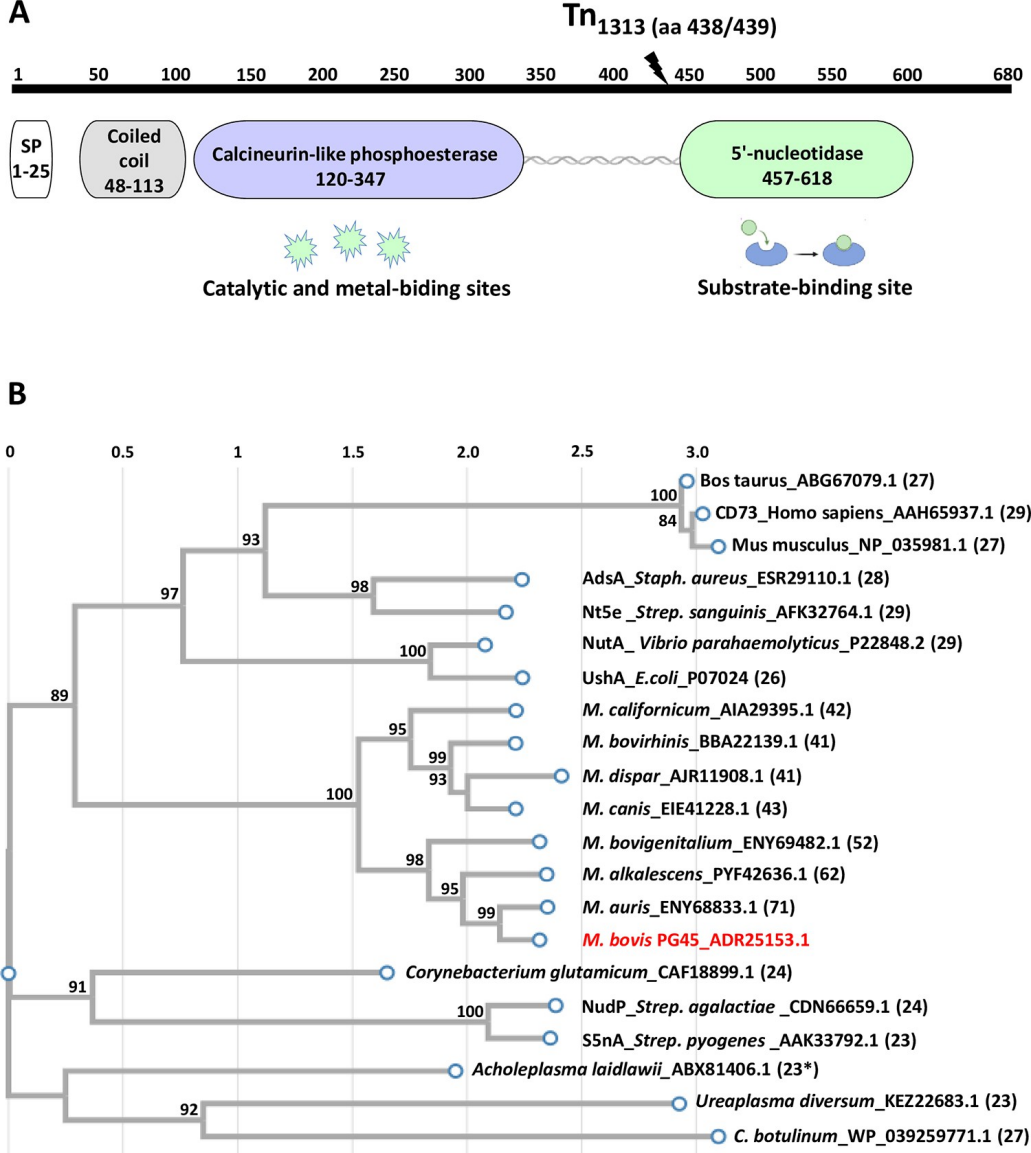
Schematic presentation *M*. *bovis* PG45 5′ nucleotidase and phylogenetic tree of its homologs. (A) Schematic diagram of the surface-exposed *M*. *bovis* 5’-NT lipoprotein is depicted based on prediction from UniProtKB software at Swiss-Prot (https://www.uniprot.org/). The diagram highlights key features, including the coiled coil (gray) sequence, calcineurin-like phosphoesterase (Metallophos, PF00149; violet), and 5′-nucleotidase (5_nucleotid_C, PF02872; green) domains. The catalytic, metal, and substrate-binding sites are schematically indicated (see also S3 Table). The grey helix signifies the region linking the calcineurin-like phosphoesterase and 5′-nucleotidase domains of *M*. *bovis* 5′-NT. The position of mTn insertion in the 5′ nucleotidase encoding gene is shown by a black arrow. SP, signal peptide sequence. The numbers on top indicate amino acid position. (B) Phylogenetic tree construction of 5′-NTs. The rooted phylogenetic tree of the *M*. *bovis* PG45 5′-NT was generated through multiple sequence alignment and phylogenetic reconstructions. ClustalW and the "build" function of ETE3 3.1.2 by Huerta-Cepas [38] were employed for these analyses, as implemented on GenomeNet (https://www.genome.jp/tools/ete/). The tree construction utilized fast tree with slow NNI and MLACC = 3 [39]. Bootstrap values are indicated at branching points, providing confidence in the tree topology. Accession numbers follow the species’ names, and the percent identity with the 5′-NT of *M*. *bovis* PG45 is presented in parentheses. *—percent identity with the 5′-NT of *A*. *laidlawii* (total 574 aa) was calculated over a 243-aa alignment only. Bovine *Mycoplasma* species without identified 5′-NT available in genomes databases (April 2024) include *M*. *alvi*, *M*. *tauri*, *M*. *testudinis*, *M*. *canadense*, *M*. *bovoculi*, *M*. *arginine*, *M*. *leachii*, *M*. *mycoides* subsp. *mycoides* SC and *M*. *wenyonii*.

While the detailed characterization of MnuA has been previously documented [15], limited information exists about 5′-NT. MBOVPG45_0690 encodes a putative surface-exposed lipoprotein spanning 680 amino acids (aa) (Fig 1A). The protein has an N-terminal signal peptide (SP; 1–25 aa) and is initially synthesized as a precursor, undergoing subsequent processing into a mature lipoprotein. The estimated molecular mass of the predicted mature protein is 73.3 kDa, with an isoelectric constant (pI) of 8.13.

*M*. *bovis* 5′-NT is a homolog of *Escherichia coli’*s UshA protein exhibiting a shared 26% identity. It belongs to the cluster of orthologous groups (COG) coding for 2′,3′-cyclic-nucleotide 2′-phosphodiesterase/5′- or 3′-nucleotidase, 5′-nucleotidase (COG0737). This protein is associated with various superfamilies, such as UshA (cl34025), metallophosphatase (cl13995), and bifunctional UDP-sugar hydrolase/5′-nucleotidase periplasmic precursor (cl35858). According to the SwissProt database (https://www.uniprot.org/), *M*. *bovis* 5′-NT displays a coiled-coil region (48–113 aa) and two domains–an N-terminal calcineurin-like phosphoesterase domain, also known as the metallophos domain (120–347 aa; Pfam ID PF00149) and a 5′-nucleotidase, C-terminal domain (457–618 aa; 5_nucleotid_C,  PF02872) (Fig 1A). Similar to UshA, the putative catalytic and metal-binding sites are located in the N-terminal domain of *M*. *bovis* 5′-NT, while the substrate-binding pocket is present in the C-terminal domain (Fig 1A); the conserved residues, identified within 5′-NT of *M*. *bovis* PG45 are listed in S3 Table. The N- and C-terminal domains of *M*. *bovis* 5′-NT are linked by a 109 aa (348–456 aa), in contrast to the 19 aa in UshA of *E*. *coli* (Fig 1A).

Within the group of bovine-related *Mycoplasma* spp., homologs to the *M*. *bovis* 5′-NT were primarily identified in species related to the same phylogenetic group, namely the Hominis group. The closest bovine-related *Mycoplasma* spp., *M*. *auris* and *M*. *alkalescens*, exhibited 71% and 62% identity, respectively to the *M*. *bovis* PG45 5′-NT. Additionally, limited but noteworthy amino acid identity was observed between *M*. *bovis* 5′-NT and nucleotidases present in the genomes of other pathogenic bacteria, as well as mammals (Fig 1B).

### Identification of *M*. *bovis* field isolates with truncated MnuA protein

To identify *M*. *bovis* strains harboring naturally truncated MnuA or 5′-NT coding genes, we performed a BLAST search analysis using the MBOVPG45_0215 and MBOVPG45_0690 genes as query sequences. Although no field strains of *M*. *bovis* with a truncated 5′-NT were identified, several had alterations in the *mnuA* encoding region, causing a shift in the translational reading frame of MnuA (S1 Fig). Specifically, two strains, 4877 (WFEJ01000016.1) and 514 (WFAW01000057.1) were identified in the cohort of Israeli isolates [40] and their *mnuA*-encoding regions were confirmed through PCR and Sanger sequencing. The *M*. *bovis* 4877 and 514 strains exhibited one (A) or four (AAAG)-base deletions within the _164_AAAAAAAAGAA_174_ region of the MBOVPG45_0215, respectively resulting in truncated proteins (S1 Fig). Furthermore, *M*. *bovis* 17DD0020 (NZ_JASFAP010000001.1, isolated in Germany [41]) with a *mnuA* sequence identical to that of *M*. *bovis* 4877, and *M*. *bovis* F9160 (CP092777.1, isolated in France [42]) containing two-bases (AG) deletion within the same region of the *mnuA* gene, leading to the truncation of MnuA were also identified (S1 Fig). Additionally, several *M*. *bovis* strains, isolated in Belgium [43], contained single or multiple changes in other regions of the *mnuA* gene, resulting in the production of abortive MnuA were identified.

### *In vitro* validation and characterization of *M*. *bovis* 0690::Tn and mnuA::Tn mutants

#### (i) The 0690::Tn mutant displayed a reduced growth rate and smaller colony size compared to the wild type

**Fig 2:**
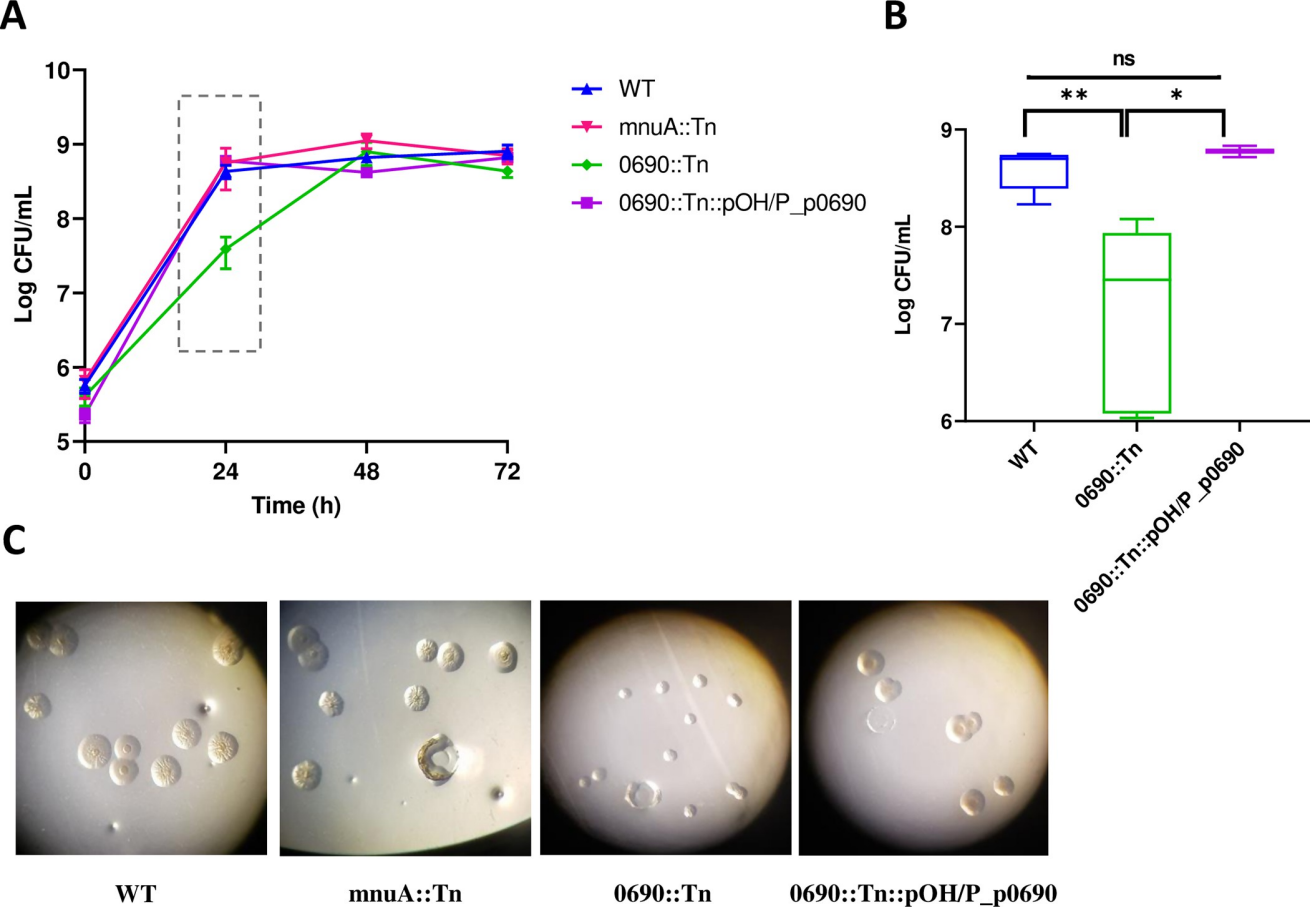
Impact of 5′ nucleotidase disruption on *M*. *bovis* growth phenotype in axenic conditions. (A) Growth curve analysis of *M*. *bovis* PG45 WT, mutants mnuA::Tn and 0690::Tn and complemented strain 0069::Tn::pOH/P_p0690 under cultivation in modified FF medium. Mycoplasma titers were determined every 24 hours over a total incubation period of 72 hours. The data are presented as the means of three independent assays, with standard deviations indicated by error bars. The hatched rectangle shows a delay in the growth rate of the 0690::Tn mutant observed during first 24 hours. (B) Statistical significance between the growth rates of the WT, the 0690::Tn and its complementation at 24-hour time point was assessed using an unpaired t-test. *P* values are indicated by asterisks (*P<0.05; **P<0.001). (C) Micrographs of *M*. *bovis* PG45 (WT), mnuA::Tn and 0690::Tn mutants, and the complemented 0069::Tn::pOH/P_p0690 colonies, grown on modified FF agar for 6 days. The images were captured under a light microscope using the same settings and magnification (x 2.5).

To assess the impact of *mnu*A and MBOVPG45_0690 disruption on the growth of mutants in axenic (modified FF) medium, we compared their growth rates and colony sizes with those of the *M*. *bovis* PG45 wild-type. The growth rate of the 0690::Tn mutant was significantly slower during the logarithmic phase, while no differences were observed between the mnuA::Tn mutant and *M*. *bovis* PG45 (Fig 2A and 2B). Additionally, the 0690::Tn mutant displayed a smaller colony size on agar compared to the WT and mnuA::Tn (Fig 2C). The growth rate as well as colony size were fully restored upon complementation of the 0690::Tn mutant using a pOH/P plasmid containing an intact MBOVPG45_0690 gene as well as its upstream and downstream noncoding regions (NCRs; 0690::Tn::pOH/P_p0690). This restoration indicates that these phenotypes are directly linked to the MBOVPG45_0690 gene (Fig 2 and S1 Data).

#### (ii) The 0690::Tn mutant exhibited significant impairment in the hydrolysis of nucleotides

**Fig 3:**
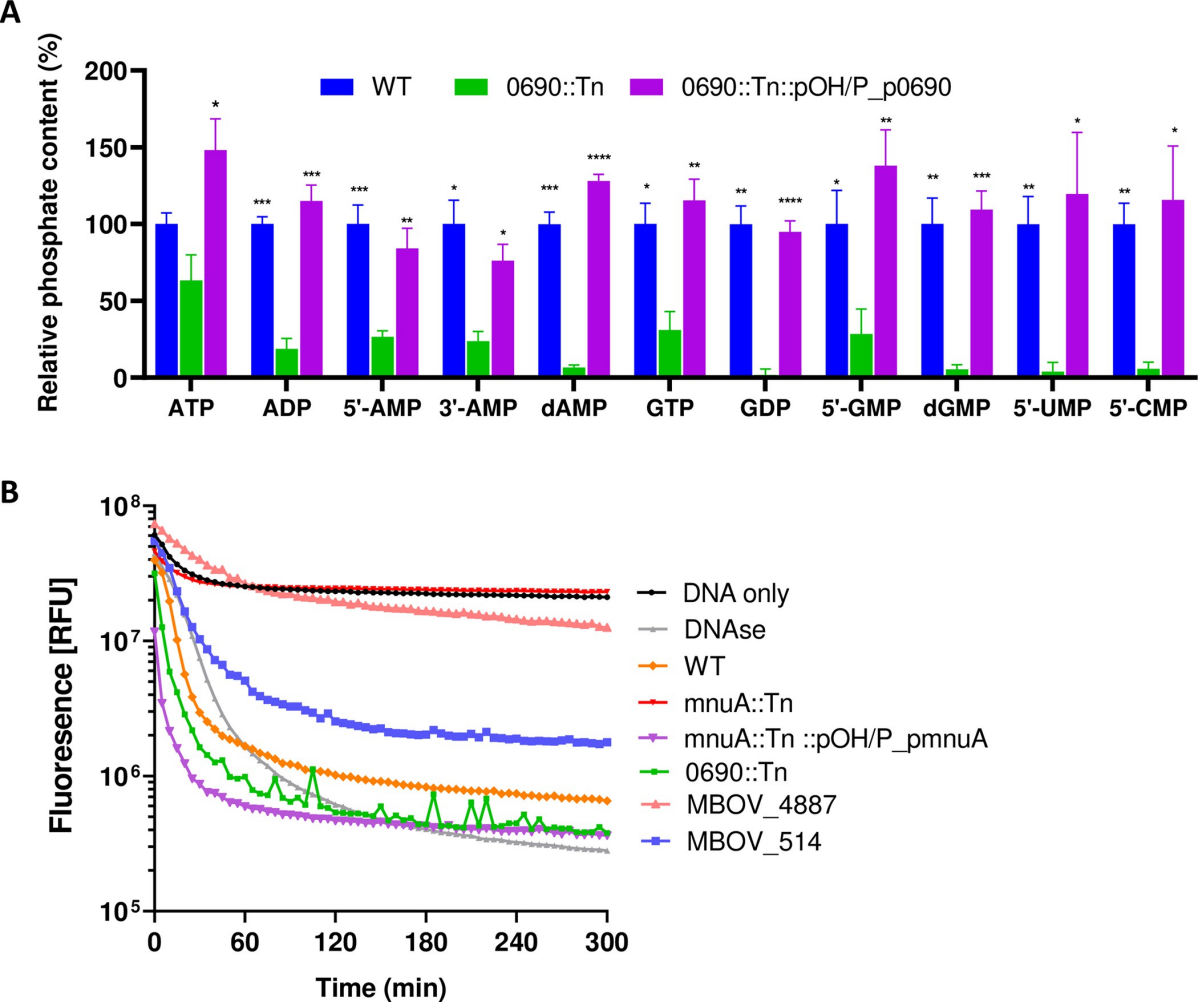
*In vitro* characterization of the mnuA::Tn and 0690::Tn mutants. (A) The hydrolytic activity of the 0690::Tn mutant towards adenosine-related nucleotides (ATP, ADP, 5′- and 3′-AMPs), guanosine-related nucleotides (GTP, GDP, 5′-GMP), as well as 5′-CMP, 5′-UMP and deoxynucleotides dAMP and dGMP was evaluated by assessing phosphate content (inorganic phosphate release) after incubation of mycoplasma cultures with the indicated substrates for 30 min at 37°C. The release of inorganic phosphate was measured using a malachite green phosphate colorimetric assay kit. The data represent the phosphate content (%) of the 0690::Tn mutant and its complemented 0690::Tn::pOH/P_p0690 strain relative to the WT. Results are derived from three independent experiments, each conducted in triplicate, and are presented as the mean ± SEM. Statistical significance was determined by an unpaired t-test. *P<0.05 **P<0.01 ***P<0.001 ****P<0.0001. (B) Nuclease activity of *M*. *bovis* Triton X-114-fractionated hydrophobic protein fractions measured by Real-time PicoGreen DNase assay. The assay conditions are described in Materials and Methods. The fluorescence signal expresses amount of the dsDNA measured over 5 hours at 37°C using the SpectraMax i3 multiple detection microplate reader. The results are represented as the mean.

To assess whether the disruption of the MBOVPG45_0690 affects the hydrolytic activity of 5′-NT, bacterial cells of *M*. *bovis* WT and the 0690::Tn mutant were incubated with a range of substrates. These included adenosine-related nucleotides (ATP, ADP, 5′- and 3′-AMPs), guanosine-related nucleotides (GTP, GDP, 5′-GMP), as well as 5′-CMP, 5′-UMP, and the deoxynucleotides dAMP and dGMP. The release of inorganic phosphate (Pi) was measured using the malachite green colorimetric assay, as described in Materials and Methods section. Compared to the WT, the total release of Pi was significantly lower for all substrates, except for ATP (*P* = 0.0775), when incubated with the 0690::Tn mutant (Fig 3A and S1 Data). Complementation of the 0690::Tn mutant restored the hydrolytic activity of *M*. *bovis* against all tested substrates (Fig 3A).

#### (iii) The mnuA::Tn mutant exhibited a loss of nuclease activity

We used real-time picogreen DNase assay to test the nuclease activity of Triton X-114 partitioned hydrophobic protein fractions of *M*. *bovis* PG45 and its two mutants in the presence of double-stranded (ds) phage λ-DNA. Particularly, we aimed (i) to validate that the mnuA::Tn mutant loss its nuclease activity; (ii) to investigate whether the 0690::Tn mutant exhibits any nuclease activity, similar to 5’-NT identified in *Streptococcus equi* subsp. *zooepidemicus* [44]; and (iii) to assess whether field isolates with truncated MnuA proteins possess reduced nuclease activity. The results revealed that both the WT and the 0690::Tn mutant efficiently degraded dsDNA as evidenced by a decrease in picogreen fluorescence. In contrast, the mnuA::Tn mutant failed to digest dsDNA (Fig 3B and S1 Data). The nuclease activity of the mnuA::Tn mutant was restored upon complementation using a pOH/P plasmid containing an intact *mnuA* gene and its NCRs (mnuA::Tn::pOH/P_pmnuA; Fig 3B). Comparison of the nuclease activity of the *M*. *bovis* 4877 and 514 field isolates, which contain truncated MnuA protein (S1 Fig) with that of *M*. *bovis* PG45 WT and the mnuA::Tn mutant revealed that while the *M*. *bovis* 514 strain demonstrated reduced nuclease activity, the *M*. *bovis* 4877 strain exhibited a loss of nuclease activity, similar to that of the mnuA::Tn mutant (Fig 3B).

### *In vivo* validation and characterization of *M*. *bovis* mutants 0690::Tn and mnuA::Tn using murine and bovine mastitis models

#### Inactivation of MnuA and 5′-NT led to reduced fitness of *M*. *bovis* PG45 in murine mammary glands

**Fig 4:**
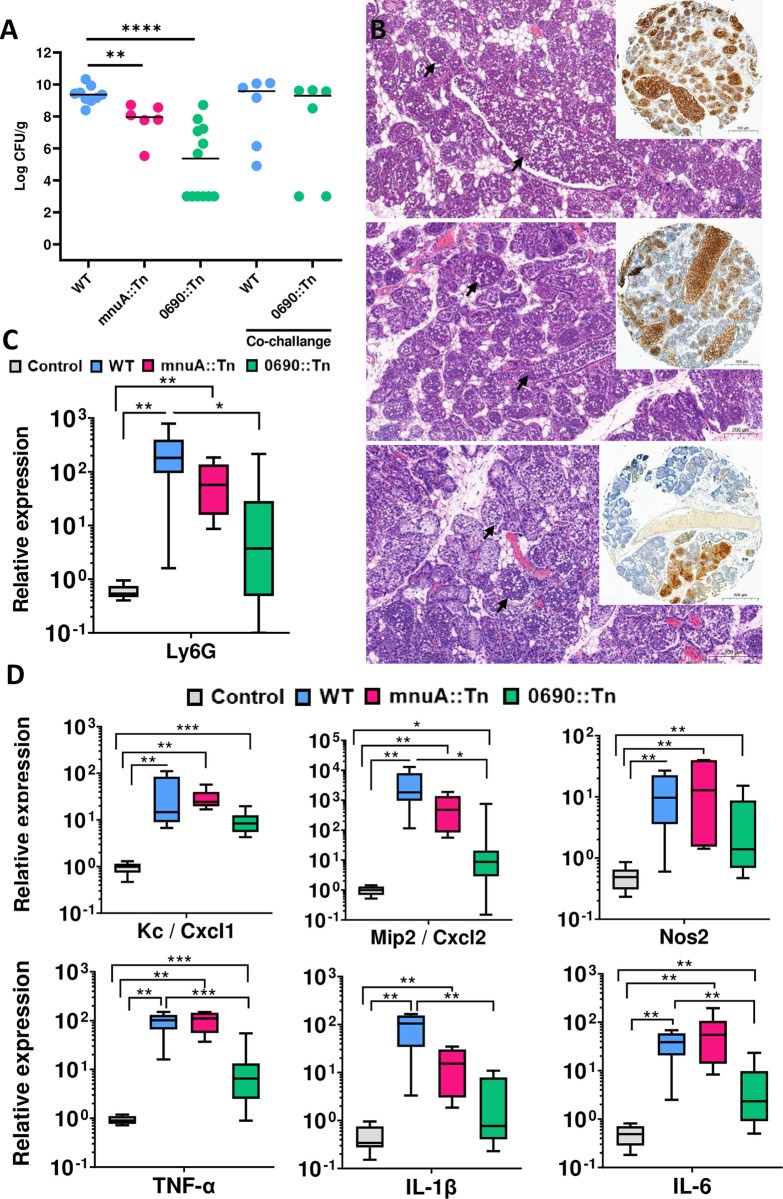
Inactivation of MnuA and 5′-NT reduced bacterial fitness and virulence of *M*. *bovis* PG45 in mammary glands. Mammary virulence of *M*. *bovis* PG45 and attenuation of the mnuA::Tn and 0690::Tn mutants were demonstrated in lactating BALB/c mice following intramammary challenge of L4 and R4 glands with approximately 10^9^ CFUs of bacteria. Bacterial colonization observed after challenge with mutant strains mnuA::Tn and 0690::Tn was significantly reduced (scatter plot in panel A), with each data point representing a single gland, and the horizontal bars indicating the median of data from three or more mice. Bacterial counts of 0690::Tn mutant were rescued following co-challenge with WT *M*. *bovis* PG45 (A). Disease manifestation was characterized by neutrophil recruitment into alveolar milk spaces (black arrows) and demonstrated in representative microscopic images of H&E and anti Ly6G (neutrophil marker) immunohistochemical staining (round insets in B) of paraffin section from WT, mnuA::Tn and 0690::Tn challenged glands (B; top, middle, and bottom panels, respectively). Using RT-qPCR, the relative expression (ΔΔC_t_) of the neutrophil marker Ly6G (C) and inflammatory marker genes MIP2, KC, Nos2, TNFα, IL1β, and IL6 (D), was quantified relative to RNA samples extracted from the mammary tissues of normal non-challenged lactating control mice. Data are presented as box plots showing higher neutrophil recruitment and expression of inflammatory markers following challenge with WT bacteria compared to the mutant strains. Statistical significance was determined by non-parametric Mann–Whitney two-independent-samples test, with a *P* value of 0.05 or less considered significant. * *P* < 0.05, ** *P* < 0.01, *** *P* < 0.001. Scale bars; 200 μm and 500 μm are shown (B).

The loss of nuclease activity in the mnuA::Tn mutant and the inability to hydrolyze nucleotides and deoxynucleotides in the 0690::Tn mutant may be associated with a loss of mammary virulence and attenuation of *M*. *bovis* PG45. To investigate this hypothesis, we utilized the recently established mycoplasma murine mastitis model in lactating mice [45,46] and compared the disease induced by *M*. *bovis* PG45 WT and the mnuA::Tn or 0690::Tn mutants (Fig 4 and S1 Data).

The results demonstrated that the inactivation of *mnu*A and MBOVPG45_0690 significantly reduced the colonization of *M*. *bovis* in the mammary gland, as evidenced by a significant reduction in bacterial counts for the mnuA::Tn and especially the 0690::Tn mutants compare to the WT (Fig 4A). Mammary gland colonization of the 0690::Tn mutant was rescued following co-challenge with *M*. *bovis* PG45 WT (Fig 4A).

Murine infected glands that developed mastitis were characterized by perfuse neutrophil recruitment into milk spaces, which was also quantified using immunofluorescence staining and relative qPCR of the lymphocyte antigen 6 complex, locus G gene (LY6G; Fig 4B and 4D). Notably, while no significant differences in the expression of inflammatory marker genes were identified in glands, challenged with the WT and mnuA::Tn mutant, the relative expression of the LY6G as well as the genes MIP2, IL1β, TNFα, and IL6 was significantly decreased in glands challenged with 0690::Tn mutant (Fig 4C and 4D). These findings support the conclusion that mnuA::Tn, but especially 0690::Tn mutants exhibited reduced bacterial fitness and attenuation in the murine mammary glands.

### The inactivation of 5′-NT led to loss of fitness and attenuated virulence of *M*. *bovis* PG45 in bovine mammary glands

To strengthen our observations in the murine model and further validate the results, we conducted a complementary *in vivo* challenge in cows using *M*. *bovis* WT and its mutants. Normal lactating cows were challenged by intramammary (IMM) infusion of *M*. *bovis* WT, and mnuA::Tn and 0690::Tn mutants. Two diagonal quarters in 3 cows were challenged with each strain or PBS as controls, while the complementary diagonal quarters were served as non-challenged controls. Clinical evaluations of cows and individual quarters were conducted using the clinical mastitis score (CMS), and milk samples were collected for California mastitis test (CMT), somatic cell count (SCC), and bacterial counts daily over a 7-day period following challenge. At the end of the study, all animals were sacrificed, and mammary tissues were sampled for histological analysis.

**Fig 5:**
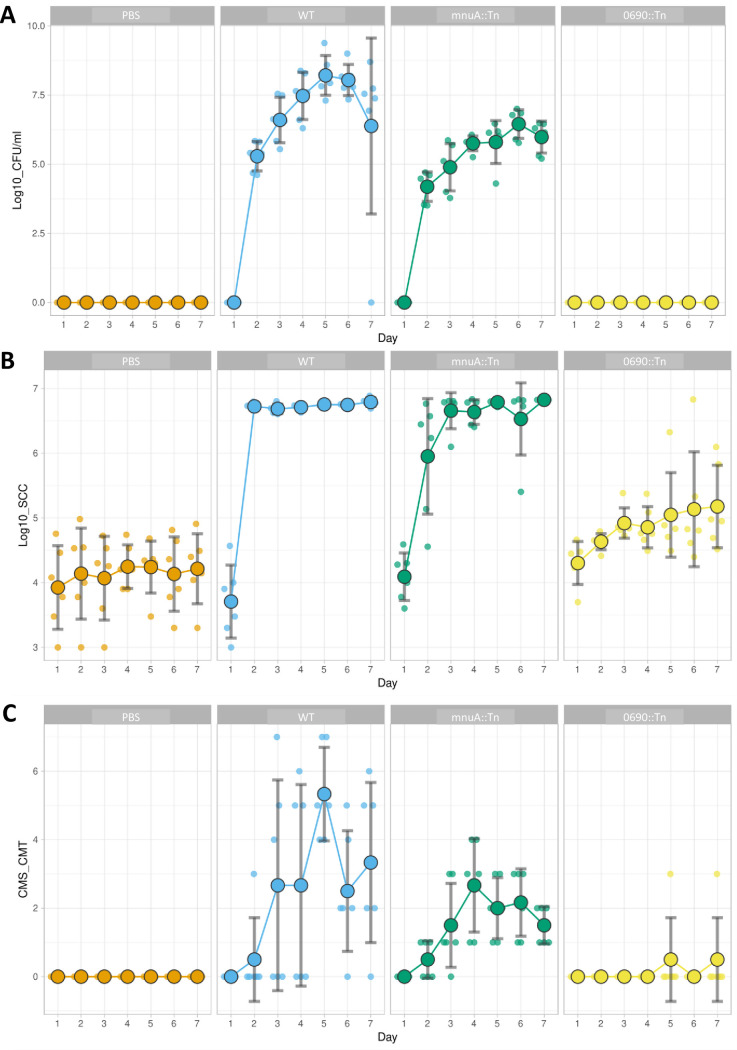
Loss of mammary fitness and virulence by *M*. *bovis* 0690::Tn mutant. Lactating cows were treated by IMM infusion with WT, mnuA::Tn, and 0690::Tn bacterial strains or PBS as non-challenged controls. Scatter plots show daily (days 1–7 after challenge) bacterial counts (CFU/ml; A), somatic cell counts (SCC; B), and individual quarter level of clinical mastitis scores (CMS; C) for each treatment in challenged glands. Daily means and SD are shown by larger dots and error bars. Plots were constructed using SuperPlotOfData [47].

**Fig 6:**
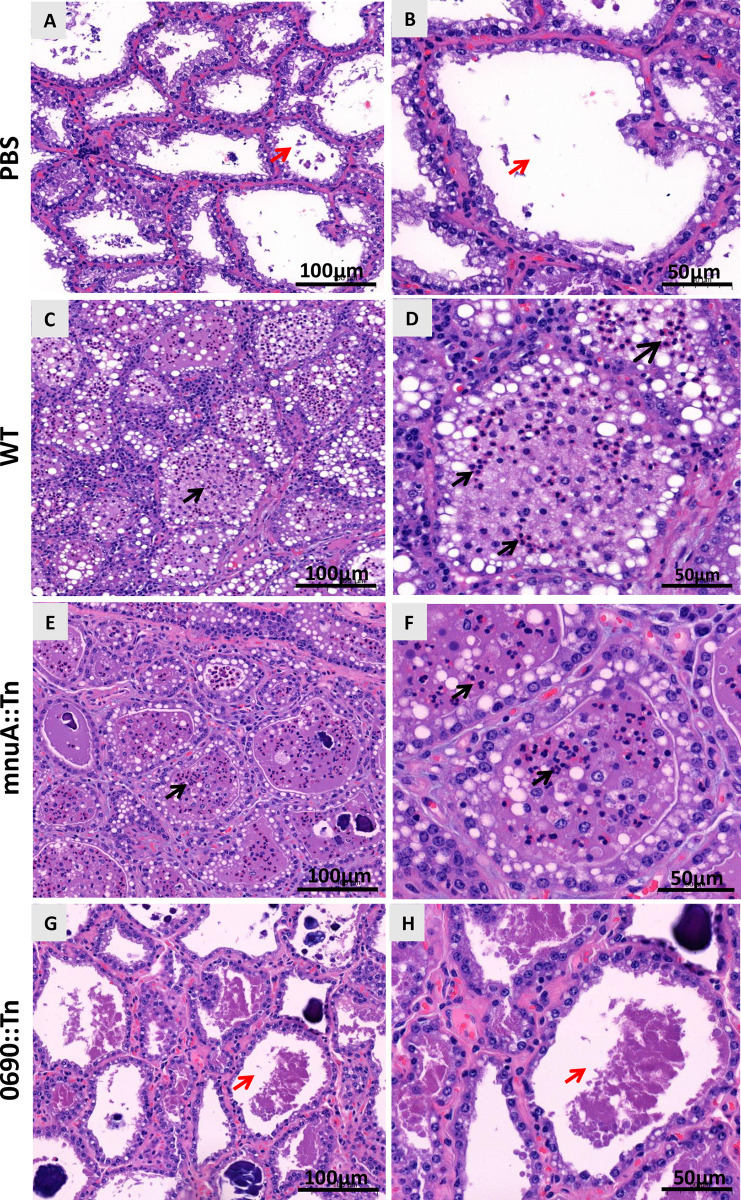
*M*. *bovis* 0690::Tn mutant failed to elicit the recruitment of neutrophils into milk spaces. H&E images of bovine mammary glands challenged with PBS (A&B), *M*. *bovis* PG45 (C&D), mnuA::Tn (E&F) and 0690::Tn (G&H) mutants. Challenge with the WT (C&D) and the mnuA::Tn mutant (E&F) elicited the recruitment of neutrophils (black arrows) into milk spaces, while immune cells populations were absent in milk spaces of mammary glands challenged with PBS (A&B) and the 0690::Tn mutant (G&H; red arrows).

Based on the bacterial counts, SCC, combined score CMS+CMT (Fig 5A–5C, respectively and S1 Data) and histological analysis (Fig 6), our results demonstrated a loss of mammary fitness and attenuation by the 0690::Tn mutant. Indeed, 0690::Tn mutant was unable to colonize the lactating bovine mammary gland, whereas the WT and mnuA::Tn mutant successfully established colonization of the challenged glands (Fig 5A) and elicited inflammatory response, characterized by high SCC, clinical mastitis (Fig 5B and 5C) and neutrophil recruitment into the alveolar and milk spaces (Fig 6).

More specifically, the combined score (CMS+CMT) in cows challenged with mnuA::Tn did not show a significant difference (P = 0.103) compared to WT-challenged cows. In contrast, cows challenged with 0690::Tn demonstrated significantly lower scores (*P*<0.001) compared to the WT-challenged group (Fig 5C). Furthermore, in comparison to the PBS-challenged group, a significant increase (*P*<0.001) in SCC over the sampling period was observed in both the WT and mnuA::Tn challenged groups. Additionally, while the SCC of cows challenged with mnuA::Tn did not exhibit a significant difference compared to the WT challenged group throughout the study period, the SCC of cows challenged with 0690::Tn was significantly lower (*P*<0.001) (Fig 5B). These results were also supported by similar bacterial counts in daily milk samples of WT and mnuA::Tn, with no growth observed for the 0690::Tn mutant strain (Fig 5A).

## Discussion

In this study, we employed genome-wide transposon mutagenesis to pinpoint and characterize mutants within MBOVPG45_0215 and MBOVPG45_0690, which encode the major membrane nuclease MnuA and 5′-NT, respectively. The MnuA and 5′-NT are lipoproteins and as such are exposed on the mycoplasma’s cell-surface, providing access to the extracellular space. The MnuA efficiently degrades nucleic acids (Fig 3B; [15]), while 5′-NT acts subsequently to hydrolyze nucleotides (Fig 3A). This enzymatic cascade generates substrates that can be transported through the cell membrane. The 0690::Tn mutant displayed a significant reduction in hydrolytic activities towards almost all nucleotides as well as dioxynucleotides tested in this study (Fig 3A). That means that *M*. *bovis* 5′-NT combines 5′-/3′-nucleosidase and nucleoside triphosphate diphosphohydrolase (NTPDase) activities, albeit the latter to a lesser extent towards ATP (Fig 3A). This mode of action aligns with observations in various bacteria, both Gram-positive and Gram-negative [20,44,48–51]. In contrast in mammals, the conversion of ATP to adenosine (Ado) requires sequential activity of two CD39 (NTPDase) and CD73 (5′-nucleotidase) enzymes [27,52]. It can be hypothesized that the ability of *M*. *bovis* 5′-NT to combine 5′/3′ nucleotidase as well as NTPDase activities provides the pathogen with a mechanism for rapid and efficient nutrient acquisition. This adaptive strategy likely contributes to the pathogen’s ability to thrive and effectively counteract the host defenses. In addition, our results show that *M*. *bovis* 5′-NT can also convert dAMP and dGMP into dAdo and dGuo, respectively (Fig 3A). It was demonstrated that dAdo, formed as a result of dAMP hydrolysis by *Staphylococcus aureus* AdsA (5′-NT), triggers caspase-3-dependent apoptosis in macrophages. This process affects phagocytic activity and facilitates the establishment of *S*. *aureus* infection [20].

Under axenic conditions, the 0690::Tn mutant displayed a slow log-phase growth and a reduced colony size, whereas the mnuA::Tn mutant exhibited growth comparable to the isogenic WT (Fig 2). At stationary phase, there was no discernible difference in bacterial counts between the WT and Δ0690, indicating the presence of alternative mechanisms for nucleotide breakdown and recycling. In a recent study, Zhu et al. [53] characterized three *M*. *bovis* proteins belonging to the DHH phosphodiesterases superfamily. These proteins demonstrated the ability to convert their substrates, either cyclic dinucleotides or nanoRNAs, into mononucleotides. Additionally, Singh et al., [54] reported the existence of an atypical class C acid phosphatase (CAPs; MBOVPG45_0528) in *M*. *bovis*. CAPs are known for their broad substrate specificity, with some acting as 5′ or 5′-3′ nucleotidases or as nicotinamide mononucleotide (NMN) 5′-NTs [55–58]. Notably, MBOVPG45_0528 is located adjacent to two DHH phosphodiesterases identified by Zhu et al., [53] suggesting their possible co-transcription. Given the absence of *de novo* nucleotide biosynthesis, the presence of multiple pathways to regulate nucleotide levels is vital for mycoplasmas.

The assessment of fitness and pathogenesis of mnuA::Tn and 0690::Tn mutants, using both murine and cow mastitis models, yielded complementary insights and mutually reinforced our findings (Figs 4–6). Disruption of 5′-NT significantly attenuates *M*. *bovis* resulting in compromised colonization (Figs 4A and 5A), decreased abilities to activate inflammatory marker genes in murine mammary glands (Fig 4B and 4C), significantly lower SSC (Fig 5B), and reduced ability to elicit inflammatory changes in milk and udder tissues in bovine mammary glands (Figs 5C and 6). Interestingly, the colonization deficiency of the 0690::Tn mutant was effectively overcome when co-infected with *M*. *bovis* PG45 WT. We hypothesize that during co-infection, the WT strain supplies essential nucleosides and other critical factors—yet to be identified—that compensate for the deficiencies of the 0690::Tn mutant, thereby restoring its ability to colonize the mammary gland (Fig 4A). In contrast to 5′-NT, the overall contribution of MnuA mutant in the pathogenesis of bovine mastitis was similar to the WT (Figs 5 and 6), and it is also consistent with the observations in the murine model (Fig 4). Circulation of *M*. *bovis* field strains, with truncated MnuA, isolated from cases of mastitis and pneumonia (S1 Fig), supports our experimental data regarding the ability of mnuA::Tn to cause mastitis.

Exonucleases and 5′-NT have been implicated in the fitness, virulence, and pathogenicity of certain bacteria [28,31]. In *S*. *aureus*, these proteins act synergistically. The nuclease degrades the DNA backbone of NETs into dNMPs. Concurrently, AdsA hydrolyzes dAMP to dAdo, resulting in macrophage cytotoxicity [20] as mentioned above. In *Streptococcus equi* subsp. *zooepidemicus*, the enzymes act in tandem possessing both nuclease and 5′-NT activities. This dual functionality enables efficient nutritional acquisition and immune evasion [44]. Moreover, surface-exposed 5′-NTs impact inflammatory and cell immune responses by regulating the ATP/Ado ratio [29,59]. Indeed, extracellular ATP and Ado are important controllers of immune cell functions acting through binding to their specific receptors present on different types of cells, including macrophages, neutrophils and dendritic cells. While extracellular ATP stimulates pro-inflammatory response, Ado induces anti-inflammatory effects on immune cells. It has been shown that e-ATP enhances mycoplasma lipoproteins–induced cytotoxicity towards different cells [60–63], while the effect of Ado remains largely unexplored. Ultimately, any disruption to the delicate ATP/Ado balance can alter the dynamics of the host-pathogen interactions, influencing the inflammatory status, disease progression, and overall outcome. It should be investigated whether *M*. *bovis* modulates the purinergic environment [59,64], and if so, whether it does so by creating an adenosine-rich, anti-inflammatory milieu that suppresses host immune responses and further supports mammary fitness and colonization. Moreover, for now, we can only speculate that the neutrophil-rich inflammatory environment elicited by the organism allows the pathogen to exploit adenosine for its benefit. Intriguingly, despite being regarded as the quintessential antibacterial cells in mammalian immunology, neutrophils seem to exacerbate pathology rather than clearing bacteria during infections with several *Mycoplasma* species, including *M*. *bovis* [65–69]. Indeed, *M*. *bovis* employs multiple strategies to evade neutrophil-mediated killing, including the inhibition of the neutrophil respiratory burst, suppression of nitric oxide and inducible nitric oxide synthase production, and avoidance of NETs-mediated trapping and killing [16,70,71].

In conclusion, our study has shown that *M*. *bovis* 5′-NT, rather than MnuA, plays a pivotal role in pathogen survival, fitness, and virulence in bovine mastitis. Further research is warranted to explore the mechanisms underlying these findings and to assess whether targeting 5′-NT gene could be a promising strategy for vaccine development and therapeutic interventions.

## Materials and methods

### Ethics statement

The IACUC approvals were obtained from the Hebrew University of Jerusalem, and the University of Tennessee with the registration numbers IACUC MD-18-15686-3 and IACUC # 2870–0322 respectively.

### Bacterial strains and growth conditions

The *M*. *bovis* PG45 type strain (ATCC 25523 / NCTC 10131 [72]) as well as two local *M*. *bovis* field isolates 514 and 4877, isolated from the milk of a dairy cow with mastitis and the lungs of a calf with pneumonia, respectively [40] were used in this study. *M*. *bovis* was propagated at 37°C in Friis (FF; [73]) modified broth medium supplemented with 0.5% (w/v) sodium pyruvate and 0.005% (w/v) phenol red (Thermo Fisher Scientific, Waltham, MA, USA). Gentamicin (100 μg/mL; Sigma-Aldrich) was added to the media for the propagation of *M*. *bovis* mutants generated by transposon mutagenesis and puromycin (5 μg/mL; Sigma-Aldrich, Rehovot, Israel) was added for the propagation of the constructs used for complementation study. Stock cultures were grown on FF-modified agar to the titers of 10^8^−10^9^ colony forming units (CFU)/mL, aliquoted, and maintained at -80°C. For each stock, the CFU/mL was determined by performing serial 10-fold dilutions in FF broth and by plating each dilution on FF agar in triplicates [74]; the agar plates were grown at 37°C, under an atmosphere of 5% CO2/95% air for 4–7 days.

### Construction of a random, genome-wide mutant collection

Transposon mutagenesis was carried out as previously described [53] using *M*. *bovis* PG45 type strain and a modified version of transposon Tn*4001* (mTn) inserted into the plasmid pMT85 [75–77]. Individual colonies of *M*. *bovis* PG45-mTn mutants were collected from several independent transformations, grown in 1 mL of selective FF medium supplemented with gentamicin and stored at −80°C in 96 well-plate format. To identify transposon insertion into specific genes (mnuA::Tn and 0690::Tn), the pools of DNA related to each 96 well plate was subjected to PCR screening using gene-specific as well as pMT85-specific primers either 195R or 3192F, located close to the 5′ and 3′ mTn-inverted repeat sequences, respectively [78]. In some cases, for example, to identify mnuA::Tn mutant, primer complementary to the upstream-located MBOVPG45_0216 gene was used. The nucleotide sequences and locations of the oligonucleotide primers are given in S1 Table. Oligonucleotide synthesis was carried out at Sigma-Aldrich (Rehovot, Israel)

PCR assays were conducted in 25 μL volumes containing 100 ng of template DNA, 0.25 μL of Phire Hot Start II DNA polymerase (Thermo Fisher Scientific) in 1× buffer supplied by the manufacturer, 0.4 μM each primer and 0.2 μM of dNTP mix. PCR amplifications were carried out in a C1000 series thermocycler (Bio-Rad, Hercules, CA, USA). The final PCR amplicons were purified using MEGAquick-spin plus total fragment PCR Purification Kit (iNtRON, Biotechnology, Gyeonggi, South Korea) and submitted to Sanger sequencing (Hylabs Ltd, Rehovot, Israel). The resultant DNA sequence was then used to identify the location of each transposon in the *M*. *bovis* PG45 genome [79] as described below.

### Illumina whole-genome sequencing and bioinformatic analyses

To confirm a single integration site of mTn insertion, whole genome sequences (WGS) of the *M*. *bovis* PG45 parental strain as well as its isogenic mnuA::Tn and 0690::Tn mutants were performed using Illumina HiSeq (Genotypic Technology Pvt. Ltd., Bangalore, India). The bioinformatic analysis was done using bioinformatic unit services at the Agricultural Research Organization (Volcani Center, Israel) using dedicated pipelines. Briefly, Illumina raw reads were subjected to quality control using FastQC software (Babraham Bioinformatics, Cambridge, UK) and low-quality reads were removed. Quality-tested and filtered reads were mapped to the reference *M*. *bovis* PG45 genome [79]. Transposon insertion sites were mapped onto the chromosome of *M*. *bovis* PG45 genome using BLAST alignment search tool for nucleotides (https://blast.ncbi.nlm.nih.gov/Blast.cgi), Geneious software version R9 (https://www.geneious.com/academic/) and DNASTAR software, version 5.06/5.51, 2003 (Lasergene Inc., Madison, WI, USA). The different functional domains were identified using SwissProt database (https://www.uniprot.org/).

### Complementation assay

Plasmid pOH/P was used as a vector for complementation studies as previously described [80] with some modifications. Briefly, for complementation of the mnuA::Tn and 0690::Tn mutants, MBOVPG45_0215 and MBOVPG45_0690 coding and noncoding regions (NCRs) positions 248826–250217 and 791010–793246 in *M*. *bovis* PG45 genome (NC_014760), respectively, were cloned into *Not*I restriction site of pOH/P plasmid (S1 Table). The resulting plasmids were transformed into *E*. *coli* strain DH10β (Thermo Fisher Scientific). Selection of the pOH/P_pmnuA and pOH/P_p0690 recombinant plasmids was done on LB plates supplemented with puromycin (5 μg/mL; Sigma-Aldrich). The pOH_pur_F1, pOH_T7_R1 or internal primers (S1 Table) were used to amplify and to sequence the inserted fragments (Hylabs). *M*. *bovis* mnuA::Tn and 0690::Tn mutants were transformed with recombinant pOH/P_pmnuA and pOH/P_p0690 plasmids, respectively to generate mutation-complemented strains mnuA::Tn::pOH/P_pmnuA and 0690::Tn::pOH/P_p0690 as previously described [80]. A single colony was selected by growing on modified FF agar containing 5 μg/mL puromycin (Sigma-Aldrich) and verified by PCR.

### Growth rate assessment of *M*. *bovis* PG45 and its mutants

Growth rates of the mnuA::Tn and 0690::Tn mutants as well complemented mutant 0690::Tn::pOH/P_p0690 were compared to the growth rate of WT *M*. *bovis* PG45 type strain. Briefly, bacterial starters (10^5^ CFU/mL) were grown in modified FF broth medium supplemented with the appropriate antibiotics at 37°C. Every 24 h, 10 μL of each culture were ten-fold diluted in FF broth, plated on FF agar and incubated at 37°C with 5% CO2/95%. The number of colonies was counted to determine the concentrations at each time point. Three independent growth trials were performed and cultures were plated in duplicates.

### Preparation of *M*. *bovis* lipoprotein fraction by Triton X-114 phase partitioning

The lipoprotein fraction of *M*. *bovis* was obtained by the TX-114 fractionation method as previously described [81]. Briefly, mycoplasma cells were pelleted from 1 L of a mid-log-phase broth culture by centrifugation at 8,000 ×*g* for 30 min at 4°, washed three times with Tris-buffered saline (TBS; Sigma-Aldrich) and resuspended in 1 mL TBS containing 1% Triton X-114 and x1 Complete Mini protease inhibitor (both from Sigma-Aldrich) at 4° for 1 h with gentle agitation. After centrifugation at 12,000 ×*g* for 30 min at 4°, the supernatant containing the soluble proteins was subjected to three cycles of phase fractionation, including incubation at 37° for 5 min for micelle formation, followed by centrifugation at room temperature for 5 min at 12,000 ×*g* for phase separation, resulting in an upper aqueous phase and a lower detergent phase containing Triton X-114 and lipoproteins. The concentration of the lipoproteins was determined by Pierce BCA Protein Assay Kit (Thermo Fisher Scientific). The lipoproteins were kept at −20° until used.

### Measurement of *M*. *bovis* nuclease activity

Nuclease activity was measured using the Quant-It PicoGreen dsDNA assay kit (Thermo Fisher Scientific) according to the manufacturer’s instructions. In brief, the total amount of lipoproteins in each well was normalized to 32 μg while the total amount of DNA in each well was normalized to 2 μg/mL by combining lysate’s DNA with λDNA (Sigma-Aldrich) in a total volume of 100 μL in a 96-well black polystyrene plate (Greiner Bio-One, Kremsmünster, Österreich). As a positive control, 1 μg/mL DNase I (Sigma-Aldrich) was added to 2 μg/mL of λDNA. PicoGreen x200 (Sigma-Aldrich, Rehovot, Israel) was diluted in a 1:200 ratio in DNase buffer, and 100 μL were added to each well. For negative control, 2 μg/mL λDNA without mycoplasmal lipoproteins or DNase I was used. The fluorescence signal was measured for 5 h at 37°C using the SpectraMax i3 multiple detection microplate reader with Ex485nm/Em530nm filter (Molecular Devices, San Jose, California, USA). Results were repeated by three independent experiments, with duplicates per lysate in each experiment.

### Measurement of *M*. *bovis* 5′-NT hydrolyze activity

Mycoplasma cells, harvested at stationary phase, were washed twice with nucleosidase buffer (50 mM Tris-HCl and 5 mM MgCl_2_; pH 7.4) and centrifuged at 10000 ×*g* for 10 min at 4°C. Pellets were resuspended in 1 mL of nucleosidase buffer containing either 1 mM of ATP, ADP, 5′-AMP, GDP, 5′-GMP, 5′-CMP, 5′-UMP, dGMP, or 0.5 mM of 3′-AMP, GTP, or 0.4 mM of dAMP (Sigma-Aldrich) to final concentration of 10^9^ CFUs/mL of bacterial cells. Reaction samples were incubated at 37°C for 30 min with shaking at 60 rpm. As controls, on one hand bacteria were incubated in nucleosidase buffer without nucleotides and on the other hand, the reaction mixture was incubated without bacteria. The reactions were stopped by adding EDTA to a final concentration of 50 mM. The reaction samples were centrifuged at 10,000 ×*g* for 5 minutes at 4°C and the supernatants were taken and diluted 4-fold with molecular biology grade water (Sartorius, Kibbutz Beit Haemek, Israel). The release of inorganic phosphate (Pi) was then quantified using a malachite green phosphate colorimetric assay kit (Sigma-Aldrich) according to the manufacturer’s instruction. In brief, 80 μL of the diluted supernatant was transferred into a 96-well plate (Corning, Corning, NY, USA), mixed with 20 μL of working reagent and incubated at room temperature for 30 min until color development. The release of Pi was measured at A620 nm and its concentration was calculated against a standard Pi curve.

### Murine mastitis model system

Mice challenge of mice was performed as previously described [45,46]. Briefly, twelve- to fourteen-week-old female BALB/c mice were used in this project (Envigo, Jerusalem, Israel). IMM challenge with ≈10^9^ CFUs /50 μL of *M*. *bovis* PG45 and its isogenic mnuA::Tn and 0690::Tn mutants was performed 8 days post-partum. IMM infusion was performed through the teat canal in both L4 and R4 abdominal mammary glands (the fourth pair found from head to tail). Three mice/six glands were used in every experiment. Mice were sacrificed 48 h post-challenge, and mammary tissues were harvested for histology, total RNA extraction and total bacterial count. Glands collected from normal non-challenged mice were used as controls. All mice were maintained under specific pathogen-free (SPF) conditions and handled under protocols approved by the Hebrew University Animal Care Committee, according to international guidelines.

### Bacterial counts and histological analysis

Mammary tissues were trisected for histology, total RNA extraction, and total bacterial count as previously described [82]. Harvested mammary tissues were weighed and homogenized in ice-cold PBS immediately after their removal, and homogenates were plated as serial 10-fold dilutions on FF-modified agar plates with or without gentamicin and bacterial colonies were counted following incubation at 37°C for 5 days, to determine the number of CFU/g of tissue. Samples for histological analysis were fixed in neutral buffered 4% paraformaldehyde (PFA) (Santa Cruz Biotechnology, Inc. Dallas, Texas, USA) and embedded in formalin-fixed paraffin-embedded (FFPE blocks), and sections were cut at a thickness of 5 μm and stained with hematoxylin and eosin (H&E) according to standard procedures. For immunohistochemistry (IHC), sections were deparaffinized and hydrated from 100% ethanol to Double Distilled Water (DDW), followed by antigen retrieval, blocking, and immunostaining with anti Ly6g (Lymphocyte antigen 6 complex locus G6D; a marker for monocytes, granulocytes, and neutrophils) antibody. Antibody binding was visualized with DAB (3,3′-diaminobenzidine) horseradish peroxidase (HRP) substrate (Vector Laboratories, ImmPACT DAB, SK-4105, Burlingame, CA, USA), followed by counterstaining with haematoxylin. The slides were dehydrated and mounted (HistoLab, PERTEX, #00801, Askim, Sweden). Images were acquired using a 3D HISTECH Pannoramic-250 microscope slide-scanner (3D HISTECH, Budapest, Hungary). Snapshots were taken with Case Viewer software (3D HISTECH, Budapest, Hungary).

### Relative quantitative Real-time PCR

Lymph node draining L4 or R4 mammary glands were dissected from unchallenged and challenged mice and RNA was extracted for qPCR and performed as previously described [45]. Briefly, total RNA was isolated from mammary tissue using the GeneElute Mammalian Total RNA Miniprep Kit (Sigma-Aldrich) combined with on-Column DNase I Digestion Set (Sigma-Aldrich). Reverse transcription was performed using qScript cDNA Synthesis Kit (Quanta BioSciences, Gaithersburg, MD, USA). PCR was conducted on a StepOne Plus PCR instrument (Applied Biosystems, Thermo Fisher Scientific) using the SYBR Green PCR Master Mix (Applied Biosystems, Thermo Fisher Scientific). All reactions were performed in triplicates and the gene expression levels for each amplicon were calculated using the ΔΔCT method [83] and normalized against prothymosin alpha encoding gene (*ptma*) mRNA.

### Intramammary challenge of dairy cows

For enrollment into the study, cows from East Tennessee AgResearch and Education Center-Little River Animal and Environmental Unit (ETREC-LRAEU) dairy herd were screened for mastitis by milk somatic cell count (SCC) and bacteriological culture weekly at 28, 21, 14 and 7 days before challenge. Twelve lactating Holstein dairy cows in their 1^st^ to 4^th^ lactations and at 25–301 days in milk that fulfilled enrolment criteria (SCC of ≤ 200,000 cells/mL of milk and negative for major bacterial mastitis pathogens and coliform bacteria) were divided into 4 groups of 3 cows each (S4 Table). Each group was transferred from the ETREC-LRAEU to the Johnson Animal Research and Teaching Unit (JARTU) at a time and given one day acclimatization before the challenge. Each animal was confined in its own pen covering 15 m^2^ area with separate feed trough and automatic freshwater bowl in the same hall with no contact with each other. Feed and water were given ad libitum following the standard protocol of ETREC-LRAEU dairy farm. Each group was monitored for 7 days post-challenge and euthanized on the 7^th^ day after clinical evaluation and sample collection. There was a one-week interval between completion of the challenge infection of one group and the beginning of the next group for proper cleaning and disinfection of the pens to avoid cross contamination among the cows and the groups.

### Challenge dose preparation and challenge infection

The challenge dose preparation was performed as described previously [84,85] with modifications. Briefly, 250 μL of 10^8^ CFU/mL of the wild-type *M*. *bovis* strain PG45 and its isogenic mutants (mnuA::Tn and 0690::Tn) were added to 5 mL of FF broth [85,86] and incubated at 37° for 24 h. The bacterial suspension was aliquoted into sterile 1 mL cryovials and stored in -80° freezer. The titers of the cultures (10^8^ CFU/mL) were determined as described above. The viability of the stock cultures at -80° was established ahead of each challenge experiment.

Before actual challenge date, 400 μL (10^8^ CFU/mL) stock culture either of wild-type *M*. *bovis* strain PG45 or the mutants, was inoculated into 40 mL FF broth and grown at 37° until mid-log phase. The growing bacterial culture was centrifuged at 17000 g for 40 min at 4°. The bacterial pellet was resuspended in 40 mL 1x PBS (pH 7.4) and centrifuged at 17000 g for 30 minutes; this step was repeated twice. The final pellet was resuspended in 40 mL sterile endotoxin-free 1x PBS. A 1 mL of *M*. *bovis* suspension at 10^8^ CFU/mL was transferred to 40 mL of sterile endotoxin free PBS. Cows received 1 x 10^6^ CFU/mL of WT, mnuA::Tn, or 0690::Tn in 5 mL of PBS into two contralateral quarters of each cow (the right front and left rear quarters). Cows in the control group were injected with 5 mL of 1x PBS (pH 7.4) in the similar manner. To determine the challenge dose, immediately after infusion the remaining bacterial suspension was plated and CFU was determined by viable colony counts as described above.

### Post-challenge health monitoring

Following the challenge, cows were monitored for 7 days for the development of mastitis or any other health problems. Cows were milked twice a day at 5 AM and 5 PM using Melasty portable milking machine (Melasty, Bursa, Turkey). To detect clinical mastitis abnormal changes in the milk and mammary glands tissue were recorded following previously described scoring system [87]. In addition, individual quarter milk samples were subjected to California Mastitis Test (CMT) (ImmuCell Corporation, Portland, Maine, USA) and SCC.

### Milk samples collection and processing

Before sample collection, teats were pre-dipped in antiseptic solution (OPI blue: dodecyl benzene sulfonic acid solution, DeLaval, Inc., Kansas, MO) and wiped with individual paper towels to remove bacteria on teat skin and teat opening. Individual quarter milk samples (3 mL) used for bacteriological analysis were aseptically collected into sterile 15 mL conical centrifuge tubes (Thermo Fisher Scientific), placed on ice, transported to the lab, and cultured immediately within 1–2 h of collection; the remaining samples were stored at -20°C until culture results were obtained.

Individual quarter milk samples were collected daily for SCC into 50 mL Capitol Vials (Thermo Fisher Scientific) tubes with preservative pills containing potassium dichromate (Capitol Vial, Auburn, AL, USA). The SCC was determined at the Dairy Herd Improvement Association (Precision Dairy Quality Laboratory, Knoxville, TN, USA) using the Soma Count 300 (Bentley Instruments Inc., Chaska, MN, USA). All udder quarters were dipped in an antiseptic solution (Bovadine: 1% iodine solution, WESTAGRO, Kansas, MO, USA) post-milking daily to prevent the spread of mycoplasma or entry of other bacterial mastitis pathogens during milking.

### Milk culture for bacteriological isolation

Bacteriological analysis was performed following National Mastitis Council (NMC) guidelines [88] with slight modification. Briefly, 100 μL of milk sample was spread onto individual FF agar plates (for mycoplasma isolation) and tryptic soy agar plates (TSA) with 5% sheep blood (for isolation microorganisms other than mycoplasma) (BD Difco, Sparks, MD, USA) and incubated at 37°C, 5% CO_2_:95% incubator for 7 days for FF and for 24 to 48 h for TSA plates. Representative mycoplasma colonies were tested by matrix assisted laser desorption ionization-time of flight mass spectrometry (MALDI-TOF) for confirmation.

### Euthanasia procedure

On the seventh day after challenge, cows were transported to a necropsy facility of Anatomic Pathology Laboratory, Department of Biomedical and Diagnostic Sciences, College of Veterinary Medicine, the University of Tennessee. Cows were sedated with intramuscular injection of xylazine at 0.73 mg/kg of body weight (100 mg/mL conc.) and euthanized with pentobarbital at 43 mg/kg of body weight (Euthasol 390 mg/mL conc.; Virbac AH, Inc., Fort Worth, TX, USA).

### Necropsy and sample collection

Samples of secretory region of mammary gland tissue were collected in 10% neutral buffered formalin (NBF) (Thermo Fischer Scientific) and submitted to the diagnostic laboratory services of the College of Veterinary Medicine, the University of Tennessee for H&E staining using their standard procedure (https://vetmed.tennessee.edu/vmc/dls/dls-forms-faqs-resources/).

### Statistical analysis

Statistical differences between *M*. *bovis* PG45, the mnuA::Tn and 0690::Tn mutants and complemented strains in growth rate, nuclease and hydrolytic activities were determined using unpaired t-test, while mean bacterial loads and genes’ relative expression (murine mastitis model) were calculated by comparing experimental groups using non-parametric Mann–Whitney two-independent-samples test. Values were first subjected to a square root transformation and did not follow a normal distribution; thus, non-parametric statistics were used. All statistical analyses were performed using GraphPad Prism 9.1.2 (GraphPad Software, Inc.), and a P value of <0.05 was considered significant.

### Experimental intramammary challenge infection in dairy cows

#### (i) Comparing challenge groups against the control group

For the analysis of quantitative outcomes (SCC, bacterial count), mixed effects linear regression with maximum likelihood estimation method was first fitted by including cow ID as a random effect. The mixed effects model was then compared against fixed effects linear regression by likelihood ratio test (LRT); when LRT was not significant, the fixed effects linear regression model was used. In all models, a full factorial design was used that evaluated the interaction between treatment and day, and their main effects. Starting with the full model, the significance of each term was assessed with the LRT by sequentially removing one term at a time. Inferences and graphs are based on the final model. The PBS group, the non-challenged quarters and the baseline (Ch+1) measurements served as references in all the analyses. Quarter SCC and *M*. *bovis* count data were log_10_ transformed and analyzed with mixed effects linear regression by including the random effect of animals and the fixed effects of treatment, day, and quarter challenge status (yes/no) and all interaction terms as full factorial analysis. Since the three-way interaction was significant for both outcomes (SCC and CFU/mL), contrasts of marginal means between two dependent variables were obtained at the levels of the third variable. Contrasts were adjusted for multiple comparisons by Sidak method. Individual quarter milk CMT scores of abnormal changes in individual quarter gland tissue, scores of abnormal changes in individual quarter milk, and mastitis were compared among the treatment groups and quarter challenge status by Fisher’s exact test.

#### (ii) Comparing between mutants and the wild-type

The log_10_ SCC and log_10_ CFU were also compared in a similar manner except that the effects were also adjusted for challenged and unchallenged quarters. To compare udder quarters’ CMT scores, individual quarter tissue abnormality scores and individual quarter milk abnormality scores, the data were binary transformed by re-categorizing scores > 0 into one category. Binary transformed data were then compared between the WT and the mutant strains either by logistic regression modelling or Fisher’s exact test, as appropriate. Similarly, quarter mastitis status was binary transformed by recategorizing the Subclinical mastitis (SCM) and Clinical mastitis (CM) cases together and analyzed with logistic regression modelling. Dairy cow challenge experiment data were analyzed using STATA 18 (StataCorp LLC, College Station, TX, USA).

## Supporting information

S1 Fig Sequence alignment of the *mnuA* genes undergo frameshift mutations in *M*. *bovis* field strains. Nucleotide sequences of the *mnuA* genes were extracted from the genomes of *M*. *bovis* PG45 type strain (ATCC 25523 / NCTC 10131; accession no. NC_014760.1) and *M*. *bovis* field strains 4877 (WFEJ01000016.1), 514 (WFAW01000057.1), 17DD0020 (NZ_JASFAP010000001.1) and F9160 (accession no. CP092777.1). The multiple alignment was conducted using the CLUSTAL O (1.2.4) (https://www.ebi.ac.uk/Tools/msa/clustalo/). The identical nucleotides are marked with asterisk (*). The start and stop codons are bolded. The region with a frameshift is marked by red, bolded and framed.
(DOCX)

S1 Table Oligonucleotides used in this study. (XLSX)

S2 Table Mapping of transposon insertions in the selected mutants of *M*. *bovis* PG45. (XLSX)

S3 Table Domain structure and conserved residues within 5′ nucleotidase of *M*. *bovis* PG45. (DOCX)

S4 Table Description of the study animals by parity and lactation cycle. (DOCX)

S1 Data Data supporting the findings of this study. (XLSX)
