# Supplementary material for: Mycoplasma bovis 5′-nucleotidase is a virulence factor conferring mammary fitness in bovine mastitis
Source: PLoS Pathog. 2024 Nov 12;20(11):e1012628. doi: 10.1371/journal.ppat.1012628 (PMC11729948; doi:10.1371/journal.ppat.1012628)
Supplement: S1 Fig — Nucleotide sequences of the mnuA genes were extracted from the genomes of M. bovis PG45 type strain (ATCC 25523 / NCTC 10131; accession no. NC_014760.1) and M. bovis field strains 4877 (WFEJ01000016.1), 514 (WFAW01000057.1), 17DD0020 (NZ_JASFAP010000001.1) and F9160 (accession no. CP092777.1). The multiple alignment was conducted using the CLUSTAL O (1.2.4) (https://www.ebi.ac.uk/Tools/msa/clustalo/). The identical nucleotides are marked with asterisk (*). The start and stop codons are bolded. The region with a frameshift is marked by red, bolded and framed. (DOCX) [file ppat.1012628.s001.docx]

***mnuA* 🡺**

**Supplementary Figure 1**

MBOV_PG45 **ATG**AAAAAGTTTAAATGAATCTATACATCTGCAGGGTTAGTTATTTCATCTAGCTCATTA 60

MBOV_4877 **ATG**AAAAAGTTTAAATGAATCTATACATCTGCAGGGTTAGTTATTTCATCTAGCTCATTA 60

MBOV_17DD0020 **ATG**AAAAAGTTTAAATGAATCTATACATCTGCAGGGTTAGTTATTTCATCTAGCTCATTA 60

MBOV_514 **ATG**AAAAAGTTTAAATGAATCTATACATCTGCAGGGTTAGTTATTTCATCTAGCTCATTA 60

MBOV_F9169 **ATG**AAAAAATTTAAATGAATCTATACATCTGCAGGGTTAGTTATTTCATCTAGCTCATTA 60

******** ***************************************************

MBOV_PG45 AGCTTTACTACTTCATGCTTTGAAAAAGGAAGTAGTAAAGAAAGTAACCAAAAAATTGTT 120

MBOV_4877 AGCTTTACTACTTCATGCTTTGAAAAAGGAAGTAGTAAAGAAAGTAACCAAAAAATTGTT 120

MBOV_17DD0020 AGCTTTACTACTTCATGCTTTGAAAAAGGAAGTAGTAAAGAAAGTAACCAAAAAATTGTT 120

MBOV_514 AGCTTTACTACTTCATGCTTTGAAAAAGGAAGTAGTAAAGAAAGTAACCAAAAAATTGTT 120

MBOV_F9169 AGCTTTACTACTTCATGCTTTGAAAAAGGAAGTAGTAAAGAAAGTAACCAAAAAATTGTT 120

************************************************************

MBOV_PG45 AAATCTAATTCAACTGATAGCACTGTAAATAAGCCATCAAAGGAAAAA**AAAG**AAGCACAA 180

MBOV_4877 AAATCTAATTCAACTGATAGCACTGTAAATAAGCCATCAAAGGAAAAA**AA-G**AAGCACAA 179

MBOV_17DD0020 AAATCTAATTCAACTGATAGCACTGTAAATAAGCCATCAAAGGAAAAA**AA-G**AAGCACAA 179

MBOV_514 AAATCTAATTCAACTGATAGCACTGTAAATAAGCCATCAAAGGAAAAA**----**AAGCACAA 176

MBOV_F9169 AAATCTAATTCAACTGATAGCACTGTAAATAAGCCATCAAAGGAAAAA**AA--**AAGCACAA 178

*********************************************** ********

MBOV_PG45 ATTAATTCTGGCAGAAGCAATGCAACAGAACACTCAGGCAACTTTAAAAACATAAATATT 240

MBOV_4877 ATTAATTCTGGCAGAAGCAATGCAACAGAACACTCAGGCAACTTTAAAAACATAAATATT 239

MBOV_17DD0020 ATTAATTCTGGCAGAAGCAATGCAACAGAACACTCAGGCAACTTTAAAAACATAAATATT 239

MBOV_514 ATTAATTCTGGCAGAAGCAATGCAACAGAACACTCAGGCAACTTTAAAAACATAAATATT 236

MBOV_F9169 ATTAATTCTGGCAGAAGCAATGCAACAGAACACTCAGGCAACTTTAAAAACATAAATATT 238

************************************************************

MBOV_PG45 GGCTTTTGAAATGTTCTAAATTACTCAAATACATCGCTTAATAAAGCATATTTTAAAACC 300

MBOV_4877 GGCTTTTGAAATGTTCTAAATTACTCAAATACATCGCTTAATAAAGCATATTTTAAAACC 299

MBOV_17DD0020 GGCTTTTGAAATGTTCTAAATTACTCAAATACATCGCTTAATAAAGCATATTTTAAAACC 299

MBOV_514 GGCTTTTGAAATGTTCTAAATTACTCAAATACATCGCTTAATAAAGCATATTTTAAAACC 296

MBOV_F9169 GGCTTTTGAAATGTTCTAAATTACTCAAATACATCGCTTAATAAAGCATATTTTAAAACC 298

************************************************************

MBOV_PG45 CAAGCGCTTGCATCTGTAATTTTTAACCAAAAATATGACTTAGTAGGGCTAGTTGAATTA 360

MBOV_4877 CAAGCGCTTGCATCTGTAATTTTTAACCAAAAATATGACTTAGTAGGGCTAGTTGAATTA 359

MBOV_17DD0020 CAAGCGCTTGCATCTGTAATTTTTAACCAAAAATATGACTTAGTAGGGCTAGTTGAATTA 359

MBOV_514 CAAGCGCTTGCATCTGTAATTTTTAACCAAAAATATGACTTAGTAGGGCTAGTTGAATTA 356

MBOV_F9169 CAAGCGCTTGCATCTGTAATTTTTAACCAAAAATATGACTTAGTAGGGCTAGTTGAATTA 358

************************************************************

MBOV_PG45 AAGGGTAGCAATAATAATCATCTTGATGAACTGATTAAACTTTTGAATGAACAAAGTGAA 420

MBOV_4877 AAGGGTAGCAATAATAATCATCTTGATGAACTGATTAAACTTTTGAATGAACAAAGTGAA 419

MBOV_17DD0020 AAGGGTAGCAATAATAATCATCTTGATGAACTGATTAAACTTTTGAATGAACAAAGTGAA 419

MBOV_514 AAGGGTAGCAATAATAATCATCTTGATGAACTGATTAAACTTTTGAATGAACAAAGTGAA 416

MBOV_F9169 AAGGGTAGCAATAATAATCATCTTGATGAACTGATTAAACTTTTGAATGAACAAAGTGAA 418

************************************************************

MBOV_PG45 AAAATCTCGTCTCATGATAGGTGGGCATACAGAGTTTCTGACAAATACATATCAAATCCT 480

MBOV_4877 AAAATCTCGTCTCATGATAGGTGGGCATACAGAGTTTCTGACAAATACATATCAAATCCT 479

MBOV_17DD0020 AAAATCTCGTCTCATGATAGGTGGGCATACAGAGTTTCTGACAAATACATATCAAATCCT 479

MBOV_514 AAAATCTCGTCTCATGATAGGTGGGCATACAGAGTTTCTGACAAATACATATCAAATCCT 476

MBOV_F9169 AAAATCTCGTCTCATGATAGGTGGGCATACAGAGTTTCTGACAAATACATATCAAATCCT 478

************************************************************

MBOV_PG45 AGTTATACAAAACATAGAGATAGTGAATTTGCAGGTTTTTTATACAAAACCAATAAATTA 540

MBOV_4877 AGTTATACAAAACATAGAGATAGTGAATTTGCAGGTTTTTTATACAAAACCAATAAATTA 539

MBOV_17DD0020 AGTTATACAAAACATAGAGATAGTGAATTTGCAGGTTTTTTATACAAAACCAATAAATTA 539

MBOV_514 AGTTATACAAAACATAGAGATAGTGAATTTGCAGGTTTTTTATACAAAACCAATAAATTA 536

MBOV_F9169 AGTTATACAAAACATAGAGATAGTGAATTTGCAGGTTTTTTATACAAAACCAATAAATTA 538

************************************************************

MBOV_PG45 GAACCTATTAAATTTAACGACGGATCTATTGGCAAAATTTACGAAAACCCTGAATTTAAG 600

MBOV_4877 GAACCTATTAAATTTAACGACGGATCTATTGGCAAAATTTACGAAAACCCTGAATTTAAG 599

MBOV_17DD0020 GAACCTATTAAATTTAACGACGGATCTATTGGCAAAATTTACGAAAACCCTGAATTTAAG 599

MBOV_514 GAACCTATTAAATTTAACGACGGATCTATTGGCAAAATTTACGAAAACCCTGAATTTAAG 596

MBOV_F9169 GAACCTATTAAATTTAACGACGGATCTATTGGCAAAATTTACGAAAACCCTGAATTTAAG 598

************************************************************

MBOV_PG45 GAAACTCCTTTTGGTGGTGGTGTTAAACACTACTCTAGGCCACCATATGCAATGAAATTC 660

MBOV_4877 GAAACTCCTTTTGGTGGTGGTGTTAAACACTACTCTAGGCCACCATATGCAATGAAATTC 659

MBOV_17DD0020 GAAACTCCTTTTGGTGGTGGTGTTAAACACTACTCTAGGCCACCATATGCAATGAAATTC 659

MBOV_514 GAAACTCCTTTTGGTGGTGGTGTTAAACACTACTCTAGGCCACCATATGCAATGAAATTC 656

MBOV_F9169 GAAACTCCTTTTGGTGGTGGTGTTAAACACTACTCTAGGCCACCATATGCAATGAAATTC 658

************************************************************

MBOV_PG45 AAAATATTAGATAGCAGTTTAAAAAATAATGATTTTACATACATTATTGACCATTTTGAT 720

MBOV_4877 AAAATATTAGATAGCAGTTTAAAAAATAATGATTTTACATACATTATTGACCATTTTGAT 719

MBOV_17DD0020 AAAATATTAGATAGCAGTTTAAAAAATAATGATTTTACATACATTATTGACCATTTTGAT 719

MBOV_514 AAAATATTAGATAGCAGTTTAAAAAATAATGATTTTACATACATTATTGACCATTTTGAT 716

MBOV_F9169 AAAATATTAGATAGCAGTTTAAAAAATAATGATTTTACATACATTATTGACCATTTTGAT 718

************************************************************

MBOV_PG45 AGTCCCGGGAAGAAAAGAGGTAACAAAGAAGTAGCTGTCAATGGTGCCGGCTCATCTGAG 780

MBOV_4877 AGTCCCGGGAAGAAAAGAGGTAACAAAGAAGTAGCTGTCAATGGTGCCGGCTCATCTGAG 779

MBOV_17DD0020 AGTCCCGGGAAGAAAAGAGGTAACAAAGAAGTAGCTGTCAATGGTGCCGGCTCATCTGAG 779

MBOV_514 AGTCCTGGGAAGAAAAGAGGTAACAAAGAAGTAGCTGTCAATGGTGCCGGCTCATCTGAG 776

MBOV_F9169 AGTCCCGGGACGAAAAGAGGTAACAAAGAAGTAGCTGTCAATGGTGCCGGCTCATCTGAG 778

***** **** *************************************************

MBOV_PG45 CTAAATGAGGCGCACAATCTCCAATACGTTTTTGACTATTTTAATGAGCTAGACGGCGAA 840

MBOV_4877 CTAAATGAGGCGCACAATCTCCAATACGTTTTTGACTATTTTAATGAGCTAGACGGCGAA 839

MBOV_17DD0020 CTAAATGAGGCGCACAATCTCCAATACGTTTTTGACTATTTTAATGAGCTAGACGGCGAA 839

MBOV_514 CTAAATGAGGCGCACAATCTCCAATACGTTTTTGACTATTTTAATGAGCTAGACGGCGAA 836

MBOV_F9169 CTAAATGAGGCGCACAATCTCCAATACGTTTTTGACTATTTTAATGAGCTATACGGCGAA 838

*************************************************** ********

MBOV_PG45 AATGATGATTTATTTTTTGCTGGAGATACTAATATTAAAGAGCGAAACCATAATGAAGCC 900

MBOV_4877 AATGATGATTTATTTTTTGCTGGAGATACTAATATTAAAGAGCGAAACCATAATGAAGCC 899

MBOV_17DD0020 AATGATGATTTATTTTTTGCTGGAGATACTAATATTAAAGAGCGAAACCATAATGAAGCC 899

MBOV_514 AATGATGATTTATTTTTTGCTGGAGATACTAATATTAAAGAGCGAAACCATAATGAAGCC 896

MBOV_F9169 AATGATGATTTATTTTTTGCTGGAGATACTAATATTAAAGAGCGAAACCATAATGAAGCC 898

************************************************************

MBOV_PG45 TTTAGCTGACTAAGCAAGAATAGCGCTTATAAAAATGTTTTTGAGCCAAACAATGAAAAT 960

MBOV_4877 TTTAGCTGACTAAGCAAGAATAGCGCTTATAAAAATGTTTTTGAGCCAAACAATGAAAAT 959

MBOV_17DD0020 TTTAGCTGACTAAGCAAGAATAGCGCTTATAAAAATGTTTTTGAGCCAAACAATGAAAAT 959

MBOV_514 TTTAGCTGACTAAGCAAGAATAGCGCTTATAAAAATGTTTTTGAGCCAAACAATGAAAAT 956

MBOV_F9169 TTTAGCTGACTAAGCAAGAATAGCGCTTATAAAAATGTTTTTGAGCCAAACAATGAAAAT 958

************************************************************

MBOV_PG45 AAGACATCACTAAGCAATACAATTGATAAATATGCTAATTCATATGACAAGATAATTCAC 1020

MBOV_4877 AAGACATCACTAAGCAATACAATTGATAAATATGCTAATTCATATGACAAGATAATTCAC 1019

MBOV_17DD0020 AAGACATCACTAAGCAATACAATTGATAAATATGCTAATTCATATGACAAGATAATTCAC 1019

MBOV_514 AAGACATCACTAAGCAATACAATTGATAAATATGCTAATTCATATGACAAGATAATTCAC 1016

MBOV_F9169 AAGACATCACTAAGCAATACAATTGATAAATATGCTAATTCATATTTCAAGATAATTCAC 1018

********************************************* *************

MBOV_PG45 CGCTCAAAGCTAAAATACATCAACCCTAAGATCTTTAAACTTTATGACTTTGTTAATAAT 1080

MBOV_4877 CGCTCAAAGCTAAAATACATCAACCCTAAGATCTTTAAACTTTATGACTTTGTTAATAAT 1076

MBOV_17DD0020 CGCTCAAAGCTAAAATACATCAACCCTAAGATCTTTAAACTTTATGACTTTGTTAATAAT 1076

MBOV_514 CGCTCAAAGCTAAAATACATCAACCCTAAGATCTTTAAACTTTATGACTTTGTTAATAAT 1076

MBOV_F9169 CGCTCAAAGCTAAAATACATCAACCCTAAGATCTTTAAACTTTATGACTTTGTTAATAAT 1078

************************************************************

MBOV_PG45 GGTTTTCTTTATAAAAATATAAATTCGATAAATGATTGAGTGCAATATGTTAAGAGCTCA 1140

MBOV_4877 GGTTTTCTTTATAAAAATATAAATTCGATAAATGATTGAGTGCAATATGTTAAGAGCTCA 1139

MBOV_17DD0020 GGTTTTCTTTATAAAAATATAAATTCGATAAATGATTGAGTGCAATATGTTAAGAGCTCA 1139

MBOV_514 GGTTTTCTTTATAAAAATATAAATTCGATAAATGATTGAGTGCAATATGTTAAGAGCTCA 1136

MBOV_F9169 GGTTTTCTTTATAAAAATATAAATTCGATAAATGATTGAGTGCAATATGTTAAGAGCTCA 1138

************************************************************

MBOV_PG45 TCAAGAAAAAAATATAGATCCGACTATGGATATATTAGAAGTGGTATTTCAGATCACTCA 1200

MBOV_4877 TCAAGAAAAAAATATAGATCCGACTATGGATATATTAGAAGTGGTATTTCAGATCACTCA 1199

MBOV_17DD0020 TCAAGAAAAAAATATAGATCCGACTATGGATATATTAGAAGTGGTATTTCAGATCACTCA 1199

MBOV_514 TCAAGAAAAAAATATAGATCCGACTATGGATATATTAGAAGTGGTATTTCAGATCACTCA 1196

MBOV_F9169 TCAAGAAAAATATATAGATCCGACTATGGATATATTAGAAGTGGTATTTCAGATCACTCA 1198

********** *************************************************

MBOV_PG45 CCAGTAGGATATACAGTTTTATTTGAA**TAA** 1230

MBOV_4877 CCAGTAGGATATACAGTTTTATTTGAA**TAA** 1229

MBOV_17DD0020 CCAGTAGGATATACAGTTTTATTTGAA**TAA** 1229

MBOV_514 CCAGTAGGATATACAGTTTTATTTGAA**TAA** 1226

MBOV_F9169 CCAGTAGGATATACAGTTTTATTTGAA**TAA** 1228

******************************

**Supplementary Figure 1. Sequence** **alignment of the *mnuA* genes undergo frameshift mutations in *M. bovis* field strains.** Nucleotide sequences of the *mnuA* genes were extracted from the genomes of *M. bovis* PG45 type strain (ATCC 25523 / [NCTC 10131](#); accession no. NC_014760.1) and *M. bovis* field strains 4877 ([WFEJ01000016.1](#)), 514 ([WFAW01000057.1](#)), 17DD0020 ([NZ_JASFAP010000001.1](#)) and F9160 (accession no. CP092777.1). The multiple alignment was conducted using the CLUSTAL O (1.2.4) ([https://www.ebi.ac.uk/Tools/msa/clustalo/](#) ). The identical nucleotides are marked with asterisk (*). The start and stop codons are bolded. The region with a frameshift is marked by red, bolded and framed.
