# Supplementary material for: Mycoplasma bovis 5′-nucleotidase is a virulence factor conferring mammary fitness in bovine mastitis
Source: PLoS Pathog. 2024 Nov 12;20(11):e1012628. doi: 10.1371/journal.ppat.1012628 (PMC11729948; doi:10.1371/journal.ppat.1012628)
Supplement: S3 Table — (DOCX) [file ppat.1012628.s004.docx]

**S3 Table. Domain structure and conserved residues within 5’ nucleotidase (5'-NT) of *M. bovis* PG45.**

| **CDC** | **Protein ID** | **Domain** | **Position of conserved domain (aa)** | **Catalytic site*** | **Metal-binding site*** | **Substrate-binding site*** | **Domain hits** |
| --- | --- | --- | --- | --- | --- | --- | --- |
| MBOVPG45_0690 | ADR25153.1 | Calcineurin-like phosphoesterase (metallophos; Pfam: PF00149) | 120-347 | D126(41) H128(43) D165(84) N197(116) H198(117) H315(217) H344(252) H346(Q254) | D126(41) H128(43) D165(84) N197(116) H315(217) H344(252) H346(Q254) | I456(R375) R460(379) R507(410) F526(429) N528(431) F606(498) D407(504) | PF00149; SSF56300 (Metallo-dependent phosphatase superfamily) |
|  |  | 5'-nucleotidase (5_nucleotid_C; Pfam: PF02872) | 457-618 |  |  |  | PF02872; SSF55816 (5'-nucleotidase (syn. UDP-sugar hydrolase), C-terminal domain) |

* The first residue number refers to *M. bovis* 5'-NT, while the second number, enclosed in parenthesis, corresponds to UshA of *E. coli* K12 (P07024).

The residues comprising the active catalytic, as well as the metal and substrate-binding sites in UshA of E. coli, have been previously reported [1-4].

**References**

1. Krug U, Patzschke R, Zebisch M, Balbach J, Strater N. Contribution of the two domains of *E. coli* 5'-nucleotidase to substrate specificity and catalysis. FEBS Lett. 2013; 587:460-466.

2. Sträter N. Ecto-5'-nucleotidase: structure function relationships. Purinergic Signal. 2006; 2:343-350.

3. Knöfel T, Sträter N. X-ray structure of the *Escherichia coli* periplasmic 5'-nucleotidase containing a dimetal catalytic site. Nat Struct Biol. 1999; 6:448-453.

4. Knöfel T, Sträter N. Mechanism of hydrolysis of phosphate esters by the dimetal center of 5'-nucleotidase based on crystal structures. J Mol Biol. 2001; 309:239-254.
