# Supplementary material for: Mycoplasma bovis 5′-nucleotidase is a virulence factor conferring mammary fitness in bovine mastitis
Source: PLoS Pathog. 2024 Nov 12;20(11):e1012628. doi: 10.1371/journal.ppat.1012628 (PMC11729948; doi:10.1371/journal.ppat.1012628)
Supplement: S4 Table — (DOCX) [file ppat.1012628.s005.docx]

**S4 Table. Description of the study animals by parity and lactation cycle.**

| Cow ID | Treatment group | Parity number | Days in milk |
| --- | --- | --- | --- |
| 1 | PBS | 1 | 202 |
| 2 | PBS | 2 | 255 |
| 3 | PBS | 1 | 159 |
| 4 | WT | 1 | 221 |
| 5 | WT | 1 | 25 |
| 6 | WT | 2 | 49 |
| 7 | mnuA::Tn | 3 | 301 |
| 8 | mnuA::Tn | 2 | 209 |
| 9 | mnuA::Tn | 1 | 174 |
| 10 | 0690::Tn | 1 | 257 |
| 11 | 0690::Tn | 3 | 252 |
| 12 | 0690::Tn | 1 | 280 |

PBS: Phosphate buffered saline

WT: wild type
